# 2,3,5,4′- Tetrahydroxystilbene-2-*O*-*β*-D-glycoside Biosynthesis by Suspension Cells Cultures of *Polygonum multiflorum* Thunb and Production Enhancement by Methyl Jasmonate and Salicylic Acid

**DOI:** 10.3390/molecules17022240

**Published:** 2012-02-22

**Authors:** Li Shao, Shu-Jin Zhao, Tang-Bing Cui, Zhong-Yu Liu, Wei Zhao

**Affiliations:** 1 School of Bioscience and bioengineering, South China University of Technology, Guangzhou 510006, China; Email: qssl@163.com (L.S.); fetbcui@scut.edu.cn (T.-B.C.); 2 General Hospital of Guangzhou Military Command, Guangzhou 510010, China

**Keywords:** *Polygonum multiflorum* Thunb, calli, suspension cells, THSG, MeJA, SA, elicitor

## Abstract

Friable calli of *Polygonum multiflorum* Thunb have been induced in MS medium supplemented with 6-benzylaminopurine (6-BA) and kinetin (KT). Suspension cultures were initiated from friable calli by inoculating calli in liquid MS medium in shake flasks in the dark and 25 °C on an orbital shaker at 100 rpm. The maximum dry weight (DW, 7.85 g/L) and 2,3,5,4′-tetrahydroxystilbene-2-*O*-*β*-D-glycoside (THSG, 56.39 mg/L) of suspension cells was obtained in MS medium after 16 days culture. Both methyl jasmonate (MeJA) and salicylic acid (SA) could increase THSG production. The most appropriate concentration of MeJA was 100 μmol/L in MS medium, in which concentration THSG content reached the maximum value of 147.79 mg/L, which represented a 162.36% increase compared to that of the control (56.33 mg/L). The most appropriate concentration of SA was 125 μmol/L in MS medium, at which concentration THSG content reached its maximum value of 116.43 mg/L, a 106.69% increase compared to that of the control (56.33 mg/L).

## Abbreviations

6-BA: 6-benzylaminopurine 2,4-D; 2,4-dichlorophenoxyacetic acid
NAA: α-naphtalene acetic acid
KT: Kinetin
HPLC: high performance liquid chromatography
THSG: 2,3,5,4′-tetrahydroxystilbene-2-*O*-*β*-D-glycoside
MeJA: Methyl jasmonate
SA: salicylic acid
DW: dry weight

## 1. Introduction

**Figure 1:**
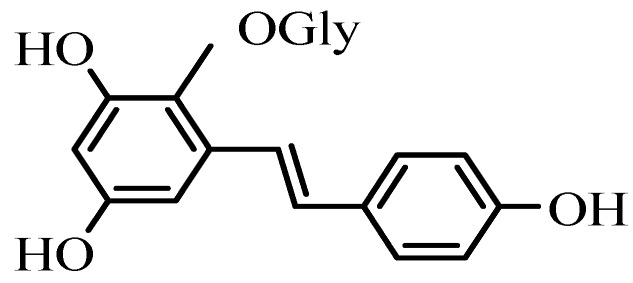
Chemical structure of THSG.

THSG (Figure 1) is the main active component of *Polygonum multiflorum* Thunb which grows in China and is commonly in Traditional Chinese Medicine. THSG serves as the quality index of *Polygonum multiflorum* Thunb according to the Chinese Pharmacopoeia, which specifies a content of not less than 1.0% (HPLC) in dried herbs. THSG possess anti-inflammatory [1,2,3] and antioxidant activity [4,5,6,7], prophylactic and therapeutic activity against Alzheimer’s disease [8,9], free radical scavenging activity [10,11], and pigmentation inducing and hair growth properties [12,13,14]. 

In general, suspension cells are the first step in the establishment of plant physiology investigations and secondary metabolite biosynthesis. For *Polygonum multiflorum* Thunb, no reports on suspension cells were published thus far. Here, the establishment of stable suspension cells of *Polygonum multiflorum* Thunb in MS medium was reported. This paved the way for further plant physiological investigation and THSG biosynthesis. MeJA and SA are commonly used as chemical elicitors to induce production of secondary metabolites in plant suspension cell cultures such as anthraquinones [15], taxane [16,17], flavonoids [18], alkaloids [19] and terpenes [20]. In the study, the enhancing effects of SA and MeJA on THSG production in suspension cells cultures of *Polygonum multiflorum* Thunb. were also investigated.

## 2. Results and Discussion

### 2.1. Friable Calli Induction and Suspension Cells Culture Establishment

**Figure 2:**
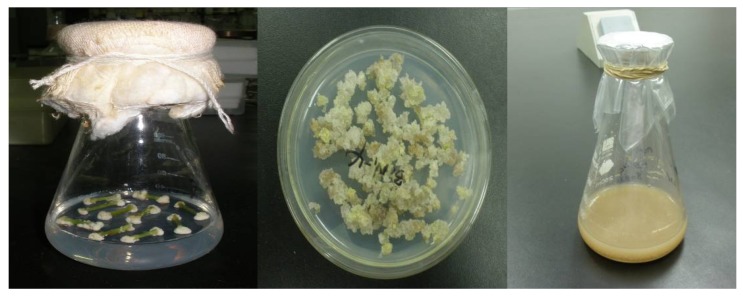
Stem calli, friable calli and suspension culture.

Calli were induced after 15 days culture. Calli were subtransferred at 15 days intervals in the same medium and culture condition. Calli were still tight after subtransferring six times, which were not suitable for suspension culture. In order to acquire friable calli, 4 g of calli were subtransferred to 40 mL of liquid MS medium which only was devoid of agar and incubated in the dark and 25 °C on an orbital shaker at 100 rpm. After 4 days, calli were then transferred to agarized MS medium supplemented with various growth regulators. By observing the friable state of calli, calli were evaluated according to browning state, growth rate and friable degree. The result showed the best friable calli can be obtained in MS medium supplemented with 1.0 mg /L 6-BA and 1.2 mg/L KT. Suspension cultures were initiated from friable calli by transferring 2 g FW cells in 250 mL shake flasks containing 60 mL of liquid MS medium in the dark and 25 °C on an orbital shaker at 100 rpm. The suspension cells were filtered through 0.6 µm sieve after 6 days. Twenty mL of supernatant was poured into 40 mL of fresh MS medium every 6 days. The cells cultures used in this study have been maintained as suspension state for over 4 months prior to experimental work (Figure 2).

### 2.2. Cell Growth and THSG Production on MS, B5 and N6 Medium

**Figure 3:**
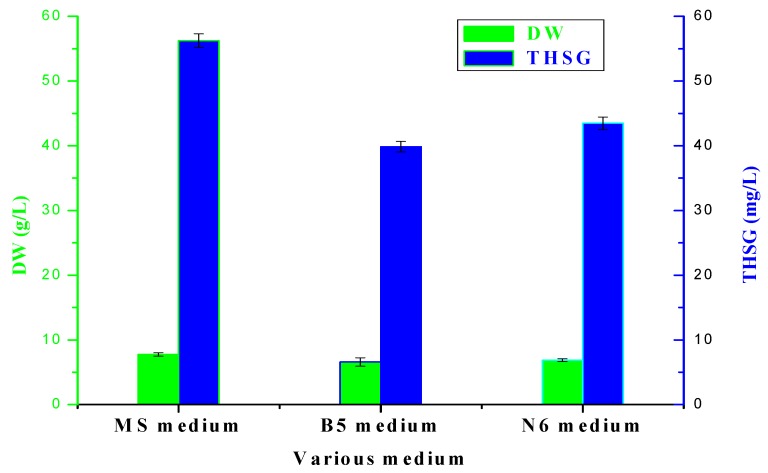
Effect of various medium on cells growth and THSG production of *Polygonum multiflorum* Thunb. FW cells were inoculated into MS medium in 250 mL shake flasks on a rotary shaker (100 rpm). The vertical bar represents standard error of three replicates.

In order to acquire the appropriate culture medium on cell growth and THSG production of *Polygonum multiflorum* Thunb, 2 g FW cells were respectively inoculated into 60 mL of MS, B5 and N6 medium supplemented with 1.0 mg/L 6-BA and 1.2 mg/L KT, after 16 days culture suspension cells were collected and analyzed which were shown in Figure 3.

The maximum DW and THSG of suspension cells, which were 7.75 g/L and 56.23 mg/L, respectively, were obtained in MS medium,. The effects of the various media on cell growth and THSG production could be explained by their different nutrient components. MS medium is more suitable for suspension cell growth than B5 and N6 medium, so MS medium was chosen as the culture medium for *Polygonum multiflorum* Thunb.

### 2.3. Time Course of Suspension Cells Cultures of *Polygonum multiflorum* Thunb

**Figure 4:**
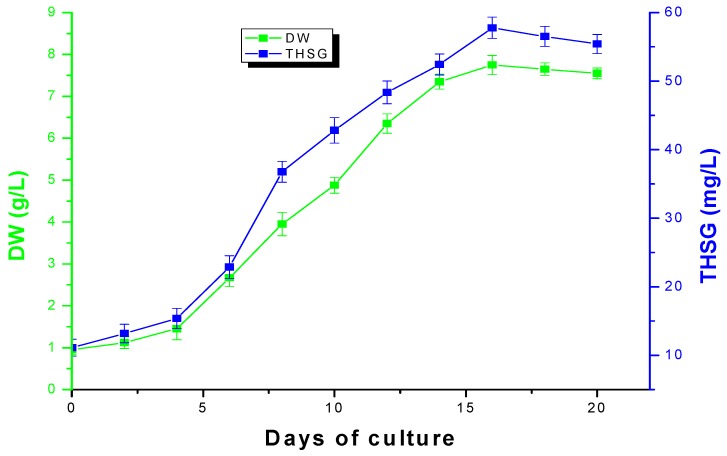
Time course of cells growth and THSG accumulation in MS medium. 2 g FW cells were inoculated into 60 mL MS medium in 250 mL shake flask on a rotary shaker (100 rpm). Vertical bar represents standard error of three replications.

The time course of cells growth and THSG production in MS medium is shown in Figure 4. The lag phase continued for 5 days, and cell growth was slow in this phase. From the 6th day to the 16th day it was in the exponential phase, and cells grew fast and THSG biosynthesis increased rapidly. The cell DW and THSG production reached their maximum values, which were 7.85 g/L DW and 56.39 mg/L, respectively, at the end of the exponential phase,. From the 17th day to the 20th day the cells were in the stationary phase, the cells DW and THSG production began to decline.

### 2.4. Different Concentrations of MeJA and SA on Cells DW and THSG Content in Suspension Cells

**Figure 5:**
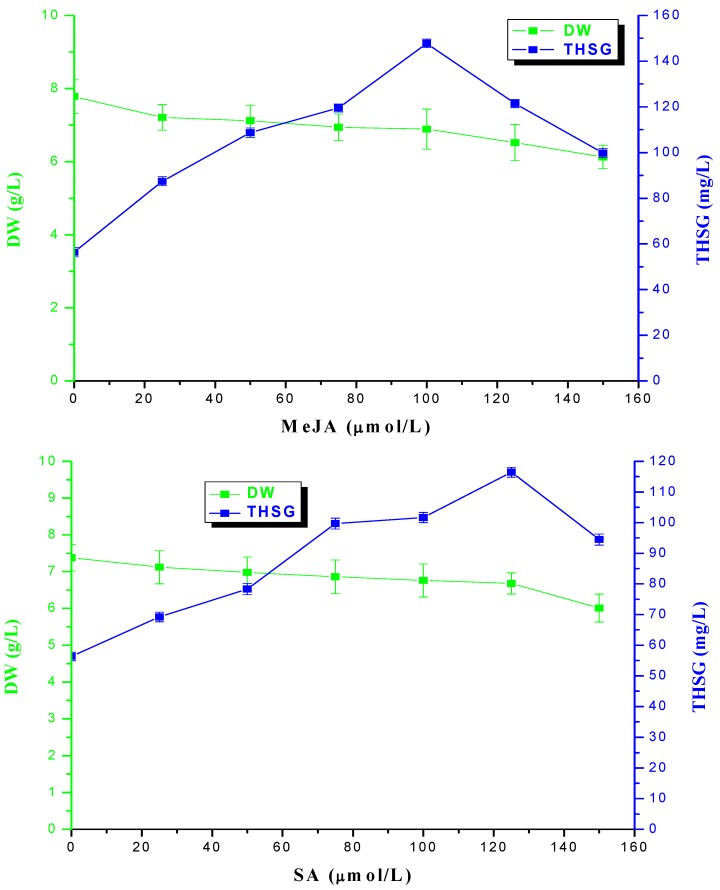
Effect of MeJA (upper) and SA (lower) concentration on DW production and THSG accumulation in MS medium. FW cells were inoculated into MS medium in a 250 mL shake flask on a rotary shaker (100 rpm) in the dark and at 25 °C. The vertical bar represents the standard error of three replicate. Elicitors were added on the 10th day of growth.

On the 10th day different concentrations of MeJA and SA were added to the MS medium, and suspension cells were harvested on the 16th day (Figure 5). From this figure, with in a certain MeJA and SA concentration range, with increasing MeJA and SA concentration, cell DWs declined and THSG content increased. When the concentration of MeJA was 100 μmol/L, THSG content reached the maximum of 147.79 mg/L (the control was 56.33 mg/L), yet cell DW declined from 7.78 g/L to 6.89 g/L. When the concentration of SA was 125 μmol/L, THSG content reached the maximum of 116.43 mg/L (the control was 56.33 mg/L), yet cells DW declined from 7.38 g/L to 6.76 g/L. With further increases of the MeJA and SA concentration, THSG content began to decline. These results showed that 100 μmol/L of MeJA had best induction effect on THSG production, and at this concentration THSG content (147.79 mg/L) was increased by 162.36% compared to that of the control (56.33 mg/L). SA at 125 μmol/L had the best induction effect on THSG production, which reached 116.43 mg/L, an increase of 106.69% compared to that of the control (56.33 mg/L).

## 3. Experimental

### 3.1. Calli Induction and Culture

Calli were induced from stem pieces of *Polygonum multiflorum* Thunb. Young stems were sterilized by immersion in 75% ethanol for 1 min, then in 0.1% HgCl_2_ for 10 min, and finally rinsed three times in sterile distilled H_2_O. Stems were cut into 0.5 cm segments. Groups of ten segments were transferred to Erlenmeyer flasks which contained 40 mL of 0.8% (w/v) agarized MS medium supplemented with 30 g/L sucrose, 0.3 g/L casein hydrolysate, 0.5 g/L proline, 1 mg/L 2,4-D and 0.4 mg/L NAA. All media were adjusted to pH 5.8 prior to autoclaving at 121 °C for 20 min. All segments were cultured in the dark at 25 °C and 90% humidity. 

### 3.2. Elicitation of Suspension Cells by MeJA and SA

MeJA and SA were dissolved in 95% ethanol at concentrations from 10 mM to 1.0 mM and sterilized by filtering through a microfilter (0.22 μm) before use. The feeding time of MeJA and SA in the medium was on 10th day. The harvest time was on the 16th day. Each treatment was repeated three times. Data were expressed as mean plus standard deviation of triplicates.

### 3.3. THSG Extraction and HPLC Analysis

Suspension cells were collected and dried at 50 °C for 12 h. Dry powder (0.5 g) was dissolved in 50% ethanol (25 mL), which were ultrasound treated three times (ice-cold), 10 min for every time. The supernatant was filtered through 0.45 μm membrane before using. THSG analysis was performed on Waters 1525 HPLC instrument (Waters, Milford, MA, USA) equipped with a binary pump, Waters 2487 dual wavelength UV detector, a manual injector and a column holder. The sample was separated on a SunFire TM C_18_ (4.6 mm× 250 mm, 5 μm, Waters) column. The mobile phase consisted of acetonitrile (CH_3_CN) and water (20:80) (vol/vol). The flow rate was 1 mL/min, and column temperature was set at 30 °C. The 2487 dual wavelength UV was monitored at 320 nm. Injection volume was 20 μL. THSG content were calculated according to the peak area and the regression equation (*y* = 26406*x* − 48094, *r^2^* = 0.9981). 

## 4. Conclusions

Friable calli can be obtained in MS medium supplemented with 1.0 mg/L 6-BA and 1.2 mg/L KT. Suspension cells cultures were initiated by inoculating 2 g FW cells into 60 mL of MS medium in 250 mL shake flasks in the dark and 25 °C on an orbital shaker at 100 rpm. The maximum DW (7.85 g/L) and THSG (56.39 mg/L) of suspension cells cultures in MS medium was obtained after 16 days of culture. THSG content of suspension cells was 7.25 ± 1.36 mg/g. THSG average synthesis rate was 453.12 μg/g·d, however THSG content of of *Polygonum multiflorum* Thunb after a year of natural growth was 29.81 ± 2.56 mg/g, the average THSG synthesis rate was 81.67 μg/g·d, showing that the synthesis rate of suspension cells was faster (5.55 times) than the natural growth. We investigated whether MeJA and SA could enhance THSG biosynthesis in suspension cells cultures of *Polygonum multiflorum* Thunb. At 100 μmol/L of MeJA in MS medium, THSG content reached the maximum value of 147.79 mg/L which corresponded to an 162.36% increase compared to control (56.33 mg/L), yet cell DW declined from 7.78 g/L to 6.89 g/L. At 125 μmol/L of SA in MS medium, THSG content reached a maximum of 116.43 mg/L, an increase of 106.69% compared to control (56.33 mg/L), yet cell DW declined from 7.38 g/L to 6.76 g/L. This showed that MeJA had better effect than SA on THSG production by *Polygonum multiflorum* Thunb.
